# Determinants of parents’ decisions on childhood immunisations at Kumasi Metropolis in Ghana

**DOI:** 10.4102/curationis.v39i1.1554

**Published:** 2016-07-29

**Authors:** Doris Hagan, Deliwe R. Phetlhu

**Affiliations:** 1 Department of Community and Health Sciences, School of Nursing, University of the Western Cape, South Africa

## Abstract

**Objective:**

To describe factors that influence parents’ decisions on childhood immunisations at Kumasi Metropolis in Ghana.

**Study design:**

Quantitative cross-sectional survey.

**Methods:**

A sample of 303 parents was obtained from a monthly accessible population of 1420 individuals from the five district hospitals through convenience sampling of respondents at immunisation sessions in Kumasi. Data obtained from the survey were analysed with SPSS version 21 software.

**Results:**

Most parents were aware of child immunisations, but they had limited knowledge on vaccines and immunisation schedules. Antenatal nurses constituted the most accessible source of vaccine information. The study established a high percentage of complete immunisation, influenced by parents’ fear of their children contracting vaccine-preventable diseases. Remarkably, some parents indicated that they immunised their children because they wanted to know the weight of their children. Forgetfulness and lack of personnel or vaccine at the centres were the reasons given by the few parents who could not complete immunisation schedules for their children, whereas the socio-demographic variables considered did not influence parents’ decision on immunisation.

**Conclusion:**

Knowledge on immunisation could not influence immunisation decisions but parents’ fear of vaccine-preventable diseases, awareness on the benefits of immunisations and sources of vaccine information were the main factors that influenced immunisation decision at Kumasi in Ghana.

## Introduction and background information

Immunisation is a recognised health preventive tool for controlling and eradicating deadly and infectious diseases (Hill & Cox 2013). It is one of the keys to achieving the fourth Millennium Development Goal (MDG 4), which is aimed at reducing under-five mortality by two-thirds by 2015 (UNAIDS 2000). Due to the effectiveness of vaccines and lessons learnt from the eradication of small pox through immunisation, the World Health Organization (WHO) established the Expanded Programme on Immunization (EPI) in 1974 to ensure that all children in all countries benefit from life-saving vaccines (WHO 2012).

Since the launch of EPI, impressive gains in vaccine coverage have been made and more children are being immunised and protected from infectious diseases compared to previous years (WHO, UNICEF, World Bank, 2009; Okwo-Béle & Cherian 2012).

Contrary to the global strides made, childhood immunisation coverage is stagnating or even declining in some areas in South Asia and large parts of Africa. About 9 million children under 5 years old are dying every year, mostly in developing countries. The global immunisation coverage for diphtheria, pertussis, tetanus (DPT3) and measles among 1-year olds in 2010 was 85%. Africa had the lowest coverage of 76%, but Ghana recorded 86% coverage, slightly above the global average but still not reaching the expected target (WHO 2012). A consistent drop of Ghana’s national immunisation coverage within the last few years has been identified in spite of diverse immunisation services provided by the country’s EPI, thereby raising questions about their effectiveness. Most of the country’s EPI interventions to improve immunisations always focus on the health worker, health systems and logistics (Ghana Health Services 2011). Factors that influence parents’ decision on childhood immunisations are mostly left out in such programmes, whereas they are the main actors in ensuring success of the programme.

Ultimately, childhood immunisation is a preventive behaviour that is directed towards the child by the parent; hence, low decision-making capacity of parents can strongly influence immunisation coverage. There have been numerous studies to examine the evidence concerning the influence of knowledge, beliefs and socio-demographic factors on child immunisation decisions in different settings (Awodele *et al*. 2010; Fantahun *et al*. 2007). Literature on previous studies revealed that factors influencing parents’ decision on childhood immunisation uptake have been argued differently by different researchers at different settings, and it is not clear which of these factors influence parents’ decision on childhood immunisations in Ghana.

The study aims to describe factors that influence parents’ decision on childhood immunisations at Kumasi Metropolis in Ghana. To achieve the aim, the Health Belief Model as a theoretical framework was used to guide the development of the study objectives which were:

- To assess parents’ knowledge on childhood immunisations and its benefits to the child.
- To identify parents’ reasons for complete and incomplete immunisation status of their children.
- To ascertain if immunisation status of children depends on the parents’ age, marital status, religion, educational level, employment status and number of children in the household.

This study was framed along the Health Belief Model developed by Hochbaum, Rosenstock and Kegels in the 1950s (Rosenstock 1990). The model establishes that the decision to access health services is motivated by the choice between one’s perceived risk of taking that action on the one hand, and the perceived benefit to be derived from accessing the service on the other. Thus the decision of parents to immunise their children is dependent on certain compelling factors such as knowledge, beliefs and individual socio-demographics. Depending on medical information, knowledge, previous experiences or beliefs, parents may perceive that VPDs are serious and their children are likely to get the disease. It is reasonable that, when people believe they are at risk for a disease, they will be more likely to seek preventive actions. Nonetheless, when they perceive that they are not at risk or have low susceptibility to a disease, decisions towards preventive actions are minimised.

In line with this, it is assumed that a perception of increased risk of a disease would lead to preventive health action, whereas perception of decreased risk of a disease would yield unhealthy actions. However, this is not always the case, as some studies have concluded that perceived risk as well as fear of harmful consequences from vaccines is highly significant in parents’ decision to immunise their children (Tickner, Leman & Woodcock 2006). Invariably, some parents are concerned about vaccine safety and immunisation side effects, whilst others are motivated to seek immunisation for their children due to fear of VPDs (McMurray *et al*. 2004). These limit the understanding of researchers on how knowledge, beliefs and perceptions as well as other social factors interact to shape parental immunisation decisions.

## Methodology

The study was conducted in Kumasi in the Ashanti Region of Ghana and the second most populous city in the country. Kumasi has numerous commercial activities compared with other cities in Ghana, which have led to its high level of urbanisation. It also has relatively low child survival rate and high fertility rate. All five district government hospitals which represented five strategic divisions of Kumasi Metropolis were selected for the data collection.

Quantitative cross-sectional survey was used to obtain information from parents of children attending the immunisation session at the selected hospitals. Based on an estimated total population of 1420 parents who visit the facilities for monthly immunisation, and considering 5% margin of error and 95% confidence level, a sample size of 303 was predetermined using Rao soft (2004) sample size calculator. Convenience sampling method was used to select 303 parents at the immunisation session.

Permission to obtain information from the health facilities was secured through the relevant authorities including the Kumasi Metropolitan Health Directorate in Ghana and all the selected hospitals. An ethical clearance letter from the Research Ethics Committee of the University of the Western Cape, copies of the participant consent form and questionnaire were provided for this purpose. Once formal permission was obtained from the relevant authorities, contacts were made with the various hospitals’ statistics departments for the necessary information regarding client attendance on immunisation sessions. Contacts were also made with the public health heads of the selected hospitals to establish appropriate dates and times to recruit respondents for the study. According to a pre-established plan, the actual data collection commenced on Monday 02 September 2013 and ended on Monday 30 September 2013.

The questionnaire was scrutinised by these experts to ensure it measured what it is intended to ‘on the face of it’ and checked if it covers the content of the construct that it was set to measure. Test–retest was also done on two occasions, 2 weeks apart at a regional hospital in Kumasi on a group of people with similar attributes as the sample population. A coefficient score of > 0.79 was obtained. Consistency of items was also checked with the use of Cronbach’s alpha.

Voluntary participation was ensured after the purpose of the study was clearly explained to the parents. They were requested to give their consent to undertake the study. Respondents who could read and write were given the questionnaire to complete, whereas a face-to-face approach was used to interview respondents who could neither read nor write. A knowledge score was generated from three sets of questions: names of vaccines, diseases against which the vaccine was given, and age at which the child received the vaccine. A simple grading system was then constructed to show the measure of knowledge level: 0–20 (poor knowledge), 21–40 (limited knowledge), 41–60 (moderate knowledge), 61–80 (good knowledge) and 81–100 (excellent knowledge).

## Analysis

The data collected were coded by assigning numerical values for identification. Quality checks were done to ensure that the questionnaires were answered as expected before being entered for analysis, using the Statistical Package for the Social Sciences (SPSS version 21). Descriptive measures were used to express the general idea of trends in the data and to show the occurrence of different observations as investigated in the study. Inferential measures were also used to infer from the sample data the relationship between immunisation status of children and the parents’ demographic data. A chi-square analysis and odds ratios at a significance level of *p* < 0.05 was done to estimate the influence of socio-demographic characteristics on immunisation of children.

## Results

All the respondents were women belonging to the reproductive age group. Majority (64.3%, *n* = 303) of them were from 21 to 30 years and 28.5% were from 31 to 40 years age groups. Only 4.9% and 0.3% were below 20 years and above 50 years, respectively. Majority (58.4%, *n* = 303) of the respondents had basic education (primary and junior high school), and about 9.8% had tertiary education, whilst 8.2% had no formal education. Most of the respondents (60.2%, *n* = 303) were self-employed, whereas 25% were unemployed. Only few (0.7%) were in general employment.

Majority had less than three children with only 13.5% (*n* = 303) having four or more children. With respect to religious background, 78.3% (*n* = 303) were Christians and 20.7% were Muslims, whereas 0.3% were Traditionalists. Almost 71% (*n* = 303) were married, whereas 29.3% were single. More than 81% (*n* = 303) of the children whose parents took part in the study were below 1 year of which 22% (*n* = 242) were below 3 months whereas only 19% were above 1 year.

### Parents’ knowledge on child immunisations

Parents’ knowledge on child immunisation was assessed with three sets of questions addressing the vaccine received during their last visit, names of diseases the children were immunised against and the age at which vaccines were given to their children. As shown in Table 1, the knowledge score for these questions was 35%.

**TABLE 1:** Knowledge level of parents.

Area of knowledge	Knowledge level (%)
Vaccine child received at the last visit (A)	31.6
Names of diseases children are immunised against (B)	22.7
Age at which vaccines are given (C)	51

**Total knowledge level (A+B+C) ÷ 3**	**35**

*Source*: Author’s own work

### Parents’ awareness on benefits of child immunisations

Parents’ awareness on the benefits of immunisation was assessed with set of questions on their sources of vaccine information, whether the sources gave enough information for their decision-making and lastly what additional information they would need for vaccine decision-making. Table 2 presents parents’ responses to these questions.

**TABLE 2:** Parents’ awareness on benefits of child immunisations.

Parents’ sources of vaccine information	*n*	%
**Where did you hear about childhood immunisations?**		
Antenatal	139	45.7
Family and friends	71	23.4
Mass media	38	12.5
Other health education	63	20.7
**Have enough information to aid you make informed decision about your child immunisation? (*n* = 301)**		
Yes	258	85.7
No	43	13.3
**If no, what additional information did you need?†**		
Information regarding risk and side effect of vaccines.	17	39.5
Information regarding benefits and effectiveness of vaccines.	23	53.5
Information on vaccine-preventable diseases and symptoms.	25	58.1
Information on when child should receive the vaccines.	14	32.6
Information on where to get the vaccine for the child.	12	27.9
Other, please specify	5	11.6

*Source*: Author’s own work

†, If the parents’ sources of vaccine information were not enough what additional information would they want to have about the vaccines their children are receiving.

### Benefits of child immunisations

Most of the respondents (302) said immunisation was beneficial because it protects children against infectious diseases. About 80%(*n* = 302) of those who said it was beneficial also indicated that it made children grow well, 71.2%(*n* = 302) opined that it made children intelligent, and 26.5%(*n* = 302) said it was beneficial because the nurses said so.

### Parents’ reasons for completing or not completing their child’s immunisation schedule

Fear of children contracting infectious diseases was the most cited reason for immunising children on time and completing the schedule. This was indicated by 238 responses constituting 78.3% of the total respondents. Other reasons included advice from family and friends (31 responses) and easy access to immunisation (13 responses). Twenty responses were from respondents who specified that they immunised their children because they wanted to know the child’s health status. Among the respondents who could not complete immunisation schedule, reasons cited included, forgetting about the next schedule date, fear of injection and absence of personnel or vaccine.

### Socio-demographic factors influencing immunisation decisions

Table 3 presents results of the bivariate analysis of socio-demographic factors influencing immunisation of children among the women involved in this study. Although there were differences in completion of immunisation with respect to the various socio-demographic groups, they could not reach significant levels.

**TABLE 3:** Results of bivariate analysis of socio-demographic factors influencing immunisation of children.

Variables	Complete immunisation schedule		*p*-value

	Yes (%)	No (%)	
**Age of respondents**			
Below 20 years	69.2	30.8	
21–30 years	85.6	14.4	0.297
31–40 years	83.5	16.5	
41 years and above	100.0	0.0	
**Level of education**			
Basic	82.9	17.1	
Secondary/vocational	87.3	12.7	0.155
Tertiary	96.4	3.6	
None	76.0	24.0	
**Employment status**			
Unemployed	82.4	17.6	
Self-employed	83.5	16.5	0.415
Working student	100.0	0.0	
General employment	92.7	7.3	
**Number of children**			
One	89.5	10.5	
Two	79.6	20.4	0.138
Three	87.7	12.3	
Four or more	78.1	21.9	
**Religion**			
Christian	86.3	13.7	
Muslim	77.8	22.2	0.192
Other	100.0	0.0	
**Marital status**			
Single	87.5	12.5	0.372
Married	83.4	16.6	

*Source*: Author’s own work

Results of the regression analysis of socio-demographic factors influencing immunisation are presented in Table 4. Respondents who were above 20 years had higher odds of completing child’s immunisation schedule, but this was not statistically significant. The various odds ratios and adjusted odds ratios and their respective confidence intervals for other variables also indicate no significant differences among the various socio-demographic groups with respect to the immunisation status of the child.

**TABLE 4:** Results of logistic regression analysis of socio-demographic factors influencing immunisation.

Socio-demographic variables	OR (95%, CI)	AOR (95%, CI)
Age of respondents (ref = below 20 years)	1.3 (0.7, 2.2)	1.6 (0.8, 3.1)
Level of education (ref = basic)	1.1 (0.8, 1.5)	1.0 (0.7, 1.4)
Employment status (ref = unemployed)	1.3 (0.9, 2.0)	1.3 (0.8, 1.9)
Number of children (ref = one)	0.8 (0.6, 1.1)	0.8 (0.5, 1.1)
Religion (ref = Christian)	0.7 (0.3, 1.3)	0.7 (0.3, 1.7)
Marital status (ref = single)	0.7 (0.3, 1.5)	0.7 (0.5, 1.1)
***N***	**-**	**299**
**Log likelihood**	**-**	**-125.018**
**Prob > Chi-squared**	**-**	**0.002**

*Source*: Au-thor’s own work

OR, odds ratio; AOR, adjusted odds ratios; CI, coefficient indicator. Main outcome = child immunisation status.

## Ethical considerations

Senate Research Committee of University of the Western Cape and the Metro Health Directorate of Ghana Health Service gave approval. Further permissions were granted by the hospitals involved according to their administrative policies. In respect of the adherence of human right principles, the following were also considered by using the approved consent form: Anonymity, Beneficence, Confidentiality, Fair treatment of respondents, Informed consent, Privacy, and Respect for persons.

## Discussion

### Parents knowledge on child immunisation

Knowledge and practices concerning immunisations are the vital contributing factors to parents’ immunisation decisions. Majority of the respondents did not know the names of the vaccines their children received at the last visit. This could limit their ability to make informed decisions on child immunisation. However, most of them had either completely immunised their children or were up to date with the immunisation schedule as recorded in previous studies (Baker, Wilson & Legwand 2007; Tarrant & Thomson 2008).

Most parents could only mention poliomyelitis and measles as the diseases the vaccines could prevent. This is because most of them knew about poliomyelitis and measles through having experienced it or seen other people affected by it. This could imply that parents who are less exposed to information on VPDs are less likely to decide for immunisation, which could lead to non-immunisation of their children. The finding is consistent with a similar study where majority of the respondents identified poliomyelitis as the primary example of vaccine-preventable diseases (Sanou *et al*. 2009).

Many of the respondents were also not sure of exact times the various vaccines were given to their children. This could be linked to some parents’ admission of forgetfulness for missing out on aspects of the immunisation schedule (Braka *et al*. 2012). It could also be deduced that their source of vaccine information did not emphasise specific times for immunisation, hence their response that they lacked information on when children received the vaccine.

### Parents’ awareness on benefits of childhood immunisations

Besides the measurement of knowledge of parents, the study also assessed parents’ awareness on the benefits of immunisation. Almost all the respondents disclosed that immunisation protected their children against infectious diseases. The assertion of this view may imply that parents have personally experienced the benefits of immunisation on their children and did not merely base their perception on information from other sources. Thus, parents have a high tendency to perceive vaccines to be good and positively decide on child immunisation to protect their children (Tomlinson & Redwood 2013; Etana & Deressa 2012).

Wrong perceptions on the benefits of the vaccine also rated highly among parents as most of them indicated that immunisation made their children intelligent and grow well. This could be due to the nutritional advice from which they benefit as part of the immunisation programme in Ghana, as well as the resultant healthy growth of their children. Whilst these wrong perceptions may have contributed to their positive response to immunisation, it establishes a knowledge gap among parents, which needs to be bridged. This gap makes them prone to different decisions when their expectations are not met. This is because in cases where their children do not portray these perceived benefits, parents may lose trust in immunisation programmes.

Most respondents in this study cited antenatal and other health educators as their source of vaccine information. Such sources may be beneficial in vaccine decision-making and influence high coverage if they address the gaps in vaccine knowledge (Adeyinka *et al*. 2009; Manjunath & Pareek 2003). Majority of the respondents disclosed that they had enough information to aid them make informed decisions about their children’s immunisation. Ironically, this high response does not correspond with their knowledge on names of the vaccines and vaccine timing. The disparity can be identified with their response on the benefits of immunisation, which revealed a high rate of misconception. As much as 80% claimed that immunisation made their children grow well, whilst 71% said it made children intelligent. Thus, the confidence displayed by parents on their knowledge of immunisation benefits was found to be misplaced, implying that they would not appreciate detailed immunisation education on vaccines and VPDs. Consequently, their decisions are likely to be inconsistent with the objectives of national immunisation programmes thereby undermining the sustainability of such programmes. The few parents who admitted that they did not have enough information to help them in vaccine decision-making indicated that they needed information on the benefits and effectiveness of vaccines, VPDs and symptoms, as well as information on side effects of vaccines. This implies that some parents may not avail their children for immunisation programmes due to ignorance of the effectiveness of the vaccines. To offset this, nurses and health educators need to find out parents’ specific information need. This would help to provide targeted information services to parents and correct prevailing misconceptions.

### Reasons parents give for completing child immunisation schedule

The reasons given by parents for completing immunisation included fear of children contracting infectious diseases, advice from family and friends, wanting to know child health status and easy access to immunisation centre. However, fear of children contracting infectious diseases was the most cited for immunising children on time and completing the schedule. Individuals who expressed fear in vaccine-preventable diseases were probably those who had previously seen a child or family member’s child afflicted with the disease. Consequently, immunisation information, which highlights the vulnerability of non-immunised children, is most likely to influence parents’ decision to immunise their children. Hence most parents immunised their children to prevent them from getting vaccine-preventable diseases (Wu *et al*. 2008). Another significant reason for immunisation was given as the advice from family and friends. This could be related to the close social ties prevalent in Ghanaian communities. Education from mass media was the least cited for complete immunisation.

Significantly, some parents specified that they immunised their children because they wanted to know their health status. This response may have been influenced by services provided during the periodic weighing of children at the facility, as part of the immunisation process. It implies that weighing services and other child health–related services that are attractive to parents would influence their decisions on immunisations and enhance coverage and sustainability, if combined with immunisation programmes.

### Reasons parents give for not completing child immunisation schedule

Forgetfulness was the most cited reason parents gave for not completing their child’s immunisation schedule. This may be due to the break between the immunisation schedule for pentavalent vaccine and the measles vaccine. It could be inferred that during this break (4th to 8th month of child’s age), parents forgot the next due date of their child’s immunisation. Also new vaccines are constantly being introduced in the EPI programme making it complex and confusing for parents to keep track of. Consequently, such parents are unable to take prompt decisions to immunise, even though they may be willing. Most mothers interviewed in similar studies cited forgetfulness as the main reasons behind incomplete immunisation status in similar studies (Abdulraheem *et al*. 2011; Jani, De Schacht & Bjune 2008; Luthy, Beckstrand & Peterson 2009). This implies that parents would require regular reminders after each immunisation visit to increase coverage.

The least cited reason was the absence of health personnel or vaccine, which led to the incomplete immunisation of their children. Similarly, some respondents in previous studies attributed their inability to achieve complete immunisation to reasons associated with health service delivery, which included lack of vaccines on the schedule date (Abdulraheem *et al*. 2011; Jani *et al*. 2008) These series of responses in the study finding show inadequate motivation for parents to complete the immunisation schedule and may account for the consistent drop in the national immunisation coverage.

### Does immunisation status of a child differ by parents’ age?

Majority of the respondents were between the ages of 21–40 years. The study found that respondents who were above 20 years had higher odds of completing child immunisation schedule. However, the differences were not statistically significant, revealing inconsistencies in the relationship between child immunisation status and the age of parents. This implies that parents’ age differences did not influence the decision to immunise their children, as their ages were not directly related to their immunisation status. Thus, irrespective of age, parents are capable of making decisions to immunise (Abdulraheem *et al*. 2011; Jani *et al*. 2008).

### Does immunisation status of a child differ by education level of parent?

The findings showed an increasing level of child immunisation with increasing educational level of mothers. Complete immunisation rate among parents with no formal education, basic education, secondary education and tertiary was, however, not statistically significant. This could be inferred that most of the respondents had positive perceptions on childhood immunisation, irrespective of their educational status. However, those with higher education were more likely to know vaccine timings, thereby adhering to the schedule. This implies that higher education will contribute more positively to vaccine decision-making. The finding is closely related to some previous studies on the continent, which also found no significant association between maternal level of education and child’s immunisation status (Bofarraj 2011).

### Does immunisation status of a child differ by parent’s employment status?

Among parents studied in both bivariate and multivariate analysis, this study revealed that parent’s occupation had no statistically significant influence on the decision to immunise children. Parents’ occupational status did not influence their decision to immunise their children. This might be related to the easy access to immunisation centres, making it feasible to immunise their children irrespective of the parent’s work schedule. As child immunisation status did not differ by parents’ occupational status, vaccine decision-making does not depend on parent’s occupation (Bofarraj 2011).

### Does immunisation status of a child differ by the number of children in a family?

This study revealed no significant relationship between parents’ number of children and the immunisation status of children. The findings also established no consistent relationship between child immunisation status and number of children. This could mean that awareness of the benefits of immunisations and fear of VPDs were strong factors influencing parents’ decision on immunisation. Contrary to this, a recent study, which accessed the immunisation coverage and its determinants among children in a peri-urban area of Kenya, found that parents with higher number of children were less likely to immunise their children (Maina, Karanja & Kombich 2013).

### Does immunisation status of a child differ by parent’s religion?

This study further reported a higher percentage of complete immunisation among respondents who were Christian than those who were Muslims. These findings show that religion influences parental decisions on immunisation. Consequently, awareness-related programmes should be more targeted at Muslim parents to enhance their decision-making on immunisation, as child immunisation status differs by religion and religion influences the willingness of parents to immunise their child (Ojikutu 2012).

### Does immunisation status of a child differ by parent’s marital status?

The marital status of mothers has also been linked to their decision to immunise children. However, this study found no significant association between marital status and child immunisation. The insignificant association may be due to the fact that both single and married parents perceived immunisations as beneficial to their children. This could be related to the high parental awareness of immunisation. Thus, parental decisions regarding immunisation are not dependent on marital status (Abdulraheem *et al*. 2011; Jani *et al*. 2008).

## Conclusion

This study concludes that parents’ knowledge on disease is not always likely to influence their decision on preventive health action but their awareness on the benefits of preventive action showed a much bigger influence on decision to take action. This gives sufficient evidence to the assumption that perceived benefits of immunisations can influence immunisation decisions. Therefore, the behaviour of parents in this study is in line with the construct of perceived benefit on the Health Belief Model, which states that a perceived benefit of health intervention is likely to increase the individual’s chance of taking the action.

The study explored the respondents’ sources of vaccine information. These sources are cues to influencing immunisation decisions. According to the Health Belief Model, preventive health behaviour is also motivated by the individual’s cues to action. Cues to action were confirmed to be effective in preventive health action as indicated in the model.

Parents perceived that vaccine-preventable diseases were severe and their children were susceptible to the diseases, hence their expression of fear of VPDs as the greatest influence on vaccine decisions. This corresponds with the construct of perceived susceptibility and perceived severity of a disease in the Health Belief Model. According to the model, the more susceptible a person feels about a disease, the greater the likelihood of taking preventive actions.

Finally, the study established that the likelihood of taking a preventive action by parents did not depend on their socio-demographic status as none of the socio-demographic variables were strong predictors of vaccine decisions. With reference to the Health Belief Model, these findings showed that socio-demographic variables considered as factors that modify a person’s perception about a disease might not be predictors to vaccine immunisation decision.

An interesting finding that emerged from this study was that some parents immunised their children because they wanted to know their health status, and not necessarily to prevent VPDs. This implies that weighing services and other child health–related services that are attractive to parents would influence their decisions on immunisations and enhance coverage and sustainability, if combined with immunisation programmes. Therefore, when planning to meet the set WHO standards on immunisation in this study context, more funds should be put towards increasing awareness on the benefits of child immunisation and other health-related services whilst reducing fear and eradicating myths about vaccines. A future study on the role of fathers in immunisation decision-making in Ghana would be revealing, as all the respondents in this study were female parents.

## Limitations

This study reflects the status of parents from only one region due to insufficient funds to cover all the 10 regions in Ghana. Again, convenience sampling limits the representativeness of the population. The results therefore need to be considered with caution as they may not be representative of all Ghanaian parents.
